# Body mass index in chronic heart failure: association with biomarkers of neurohormonal activation, inflammation and endothelial dysfunction

**DOI:** 10.1186/1471-2261-13-80

**Published:** 2013-10-01

**Authors:** Heidi M Christensen, Morten Schou, Jens P Goetze, Jens Faber, Jan Frystyk, Allan Flyvbjerg, Caroline Kistorp

**Affiliations:** 1 Departments of Cardiology and Endocrinology, Herlev University Hospital, Ringvej 75, Herlev 2730, Denmark; 2 Department of Cardiology, Rigshospitalet, University of Copenhagen, Copenhagen, Denmark; 3 Department of Clinical Biochemistry, Rigshospitalet, University Hospital of Copenhagen, Copenhagen, Denmark; 4 Department of Endocrinology, Herlev University Hospital, Herlev, Denmark; 5 Faculty of Health Sciences, Copenhagen University, Copenhagen, Denmark; 6 Department of Endocrinology and Internal Medicine, Aarhus University Hospital, Aarhus, Denmark; 7 The Medical Research Laboratories, Department of Clinical Medicine, Faculty of Health, Aarhus University, Aarhus, Denmark

**Keywords:** Biomarkers, Chronic heart failure, Body mass index

## Abstract

**Background:**

Low body mass index (BMI) is associated with a poor outcome in chronic heart failure (CHF). An inverse association between BMI and adiponectin and N-terminal pro-B-type natriuretic peptide (NT-proBNP) has been reported. The aim of the present study was to investigate whether novel markers of neurohormonal activation, inflammation, and endothelial dysfunction are associated with BMI in CHF.

**Methods:**

In a cross-sectional study including 171 patients with CHF and a left ventricular ejection fraction (LVEF) ≤45% the impact of BMI on circulating plasma concentrations of adiponectin, α-defensins, high sensitivity C-reactive protein (hsCRP), copeptin, mid-regional pro-adrenomedullin (MR-proADM), NT-proBNP, and mid-regional pro-A-type natriuretic peptide (MR-proANP) were evaluated.

**Results:**

In multivariable linear regression analysis including age, sex, LVEF, New York Heart Association functional classification (NYHA), estimated glomerular filtration rate (eGFR), and diabetes, only NT-proBNP (β = −0.32) and adiponectin (β = −0.39) remained independently associated with BMI. MR-proANP was associated with BMI but adjusting for age attenuated the relation being no longer significant.

**Conclusions:**

Among biomarkers typically increased in patients with CHF only adiponectin and NT-proBNP demonstrated independent inverse associations with BMI. This indicates a direct effect of these two biomarkers enhancing the wasting process seen in CHF.

## Background

Low body mass index (BMI) is associated with a poor outcome in chronic heart failure (CHF) and survival is further impaired if CHF progresses to cardiac cachexia [1,2]. The underlying mechanisms are still undergoing intense investigations. Interestingly, a role of the failing heart in regulation of adipose tissue has been suggested [3]. Concentrations in plasma of the cardiac natriuretic peptides (NPs), atrial (ANP) and B-type (BNP), and the N-terminal fragment of the pro-hormone pro-BNP (NT-proBNP), are markedly increased in CHF and related to impaired outcome [4]. Previous studies have shown that BNP and NT-proBNP concentrations are inversely associated with BMI, and particularly high levels have been reported in patients with cardiac cachexia [5-7]. The mid-regional sequence of proANP (MR-proANP) has emerged as a novel biomarker of survival in CHF. Available data assessing the relationship with BMI have not been conclusive and have mainly been addressed in conditions with acute HF [8,9]. Furthermore, in previously trials ANP has been reported to be less influenced by BMI, and the impact on low BMI in patients with CHF has not yet been clarified.

High levels of the adipocytokine adiponectin reflect low BMI as well as high insulin sensitivity in healthy subjects and patients with type 2 diabetes mellitus (DM). Although adiponectin correlates to BMI in CHF, a paradox seems to be present, since high adiponectin levels have been reported to be associated with increased risk of mortality in CHF [6,10,11], and further reported elevated in patients with cardiac cachexia, irrespectively of BMI [12].

The development of novel assays targeting pro-hormones and stable fragments of peptides associated with cardiovascular disease (CVD) presents several new biomarkers with a potential for clinical use. Adrenomedullin (ADM) is a 52-amino-acid peptide that is expressed in various tissues and its secretion is stimulated in CHF where high concentrations reflect poor survival [13]. Circulating levels of the stable mid-regional fragment of the pro-hormone pro-adrenomedullin peptide (MR-proADM) is associated with BMI in obese patients without heart disease, and decline during weight reduction after gastric bypass surgery [14]. Copeptin is excreted in equimolar amounts to arginine vasopressin (AVP), as the C-terminal of the pro-hormone by the posterior pituitary gland, in response to reduced osmolality and hypovolemia. In CHF copeptin concentrations are elevated and associated with increased risk of mortality [15]. Previous CHF studies of the prognostic value of copeptin and MR-proADM have to the best of our knowledge, not addressed the effect of BMI in details.

Increased low-grade inflammation is an important characteristic in CHF [16]. The traditional inflammatory biomarker used in various clinical settings is high sensitivity C-reactive protein (hsCRP). High levels of hsCRP are seen in healthy individuals with high BMI, and moreover, weight loss has been associated with decreasing hsCRP [17]. The recently described inflammatory biomarker, α-defensins, is elevated in CHF patients and associated with increased risk of all-cause mortality [18]. The relationships between circulating levels of these biomarker and BMI have not been evaluated in earlier CHF studies.

We have previously reported of an association between BMI and NT-proBNP as well as adiponectin in CHF [6]. However, whether low BMI has an impact on plasma concentrations of these biomarkers is unknown. Therefore, we aimed to investigate the association between the biomarkers examined and BMI in patients with CHF.

## Methods

### Study sample

From 2000 through −2003, we enrolled 171 patients with confirmed systolic CHF defined as left ventricular ejection fraction (LVEF) ≤45% by echocardiography in combination with symptoms. All patients were recruited at a specialized CHF clinic at Frederiksberg University Hospital, Copenhagen, Denmark. Data on BMI and biomarkers were available in 171 patients. The original design of the cohort study as well as baseline characteristics have been published previously [19]. The investigations conformed to the principles outlined in the Declaration of Helsinki. The study was approved by the local Ethics Committee and all patients gave written informed consent.

### Laboratory measurements

All patients met at the outpatient clinic for blood sampling following an overnight fast (8 hrs). Venous blood was drawn and stored as EDTA-plasma at −80°C in aliquots until analysis. A urine sample was collected. To determine the plasma level of α-defensins we used a validated, in-house, solid-phase RIA. Using microtiter plates (from Nunc, Roskilde, Denmark) incubated overnight at 5 C with anti-mouse IgG (Sigma-Aldrich, Copenhagen, Denmark). After washing, all wells were added 50 μL of standard (purified α-defensins-1, Sigma-Aldrich) or diluted plasma (1 in 25), 50 μL of ^125^I-labeled α-defensins (∼10.000 cpm) and 100 μL (200 μg/L) of a specific monoclonal antibody, which recognizes α-defensins-1, -2, and −3 (clone DEF 3, BMA, Augst, Switzerland). The intra- and inter-assay coefficients of variation (CVs) were 6% and 9%, respectively. Repetitive freezing and thawing (nine cycles) on serum and plasma levels of α-defensins did not affect the α-defensins levels [20]. All blood samples were measured within the same assay-run. HsCRP was measured with a latex-particle-enhanced immunoassay (Roche Diagnostics, Germany). Plasma NT-proBNP was measured by using a highly sensitive and specific immunoassay based on double-antibody sandwich technique (Roche Diagnostics, Mannheim, Germany) [21]. To determine plasma level of total adiponectin we used a validated, in-house, time resolved immunofluorometric assay (TR-IFMA) based on commercially available antibodies and recombinant human adiponectin obtained from R&D Systems, Abingdon, UK. The detection limit is <1.5 μg/l. The within-assay coefficient of variation (CV) of standards and unknown samples averaged <5%; the in-between assay CV was 8-12% depending on concentrations of adiponectin [22]. Plasma concentrations of midregional proANP (MR-proANP), MR-proADM, and copeptin, were measured on the Kryptor Compact platform (B-R-A-H-M-S, Henningsdorf, Germany) [23-25].

### Statistical analysis

We categorized patients according to BMI levels ≤21 kg/m^2^ 21–25 kg/m^2^, and >25 kg/m^2^. The cut point of BMI of 21 kg/m^2^ was pre-specified and used to facilitate comparison with previous studies in patients with cardiac cachexia [1]. We used one way ANOVA for comparing data with a normal distribution and a Kruskal-Wallis test for non-parametric data. Parameters with a skewed distribution (adiponectin, NT-proBNP, MR-proANP, MR-proADM, copeptin, α-defensins, and hsCRP) were ln transformed prior to linear regressions and log (2) transformed prior to Cox proportional hazard analyses. Hazard ratio (HR) for transformed data was expressed per doubling in the respective biomarker. In multivariable linear regression analyses, we examined the association between BMI as the dependent variable and biomarkers adjusted for age, sex, LVEF, New York Heart Association functional classification (NYHA), estimated glomerular filtration rate (eGFR), and DM. All values are 2 tailed, and p- values below 0.05 were considered statistically significant. The statistical software package SPSS version 20.0 was used for all analyses.

## Results

### Baseline characteristics

Clinical characteristics according to BMI are presented in Table 1. The patients were older in the lower BMI categories, they had the lowest prevalence of DM, and had the highest levels of circulating adiponectin, NT-proBNP, and MR-proANP. A low BMI (<21 kg/m^2^) was associated with markedly elevated hsCRP concentrations. Fifty-seven% of the patients had ischemic heart disease (IHD).

**Table 1:** Comparison of clinical characteristics according to BMI categories

**BMI**	**≤21 kg/m**^**2**^ **N = 11**	**21-25 kg/m**^**2**^ **N = 52**	**>25 kg/m**^**2**^ **N = 108**	**P-value**
**Clinical characteristics**				
Age, years	74 (10)	70 (11)	67 (10)	0.04¶
Sex, female %	46	37	20	0.04†
eGFR, ml/min/m^2^	77 (31)	66 (29)	74 (31)	0.27¶
CHF Duration*, months	1.5 (1–69)	3 (2–30)	6 (3–36)	0.17†
LVEF, %	34 (8)	30 (9)	30 (8)	0.35¶
NYHA class I/II, %	82	73	73	0.81†
Type 2 Diabetes Mellitus, %	9	15	33	0.02†
P-sodium, mmol/l	138 (3)	137 (4)	138 (3)	0.35¶

P-value is a comparison of mean (¶ = ANOVA)/ median/percentages († = Kruskal Wallis Test/Chi2 Test) between patients with BMI ≤ 21, 21–25 and BMI >25 kg/m2.

Mean (SD), * = median (interquartile range); ¶ = ANOVA; † = Pearson chi2 test/Kruskal Wallis Test.

*Abbreviations:* BMI: body mass index, CHF: chronic heart failure, eGFR: estimated glomerular filtration rate, LVEF: left ventricular ejection fraction.

### Correlations between biomarkers

Both inflammatory biomarkers correlated to MR-proADM (hs-CRP: β = 0.27 and α-defensins: β = 0.36, p < 0.0001 for both). Adiponectin concentrations were positively associated with MR-proANP (β = 0.36, p < 0.0001).

### Association between BMI and biomarkers

**Figure 1:**
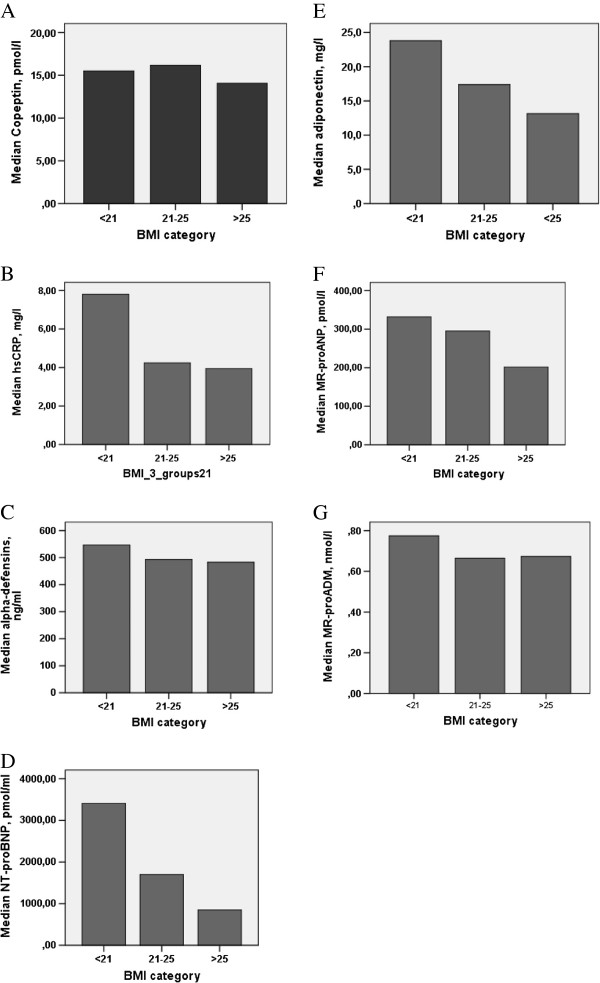
**Biomarkers according to BMI categories.** Legend: Median concentrations of Copeptin **(A)**, hsCRP **(B)**, α-defensins **(C)**, NT-proBNP **(D)**, Adiponectin **(E)**, MR-proANP **(F)** NT-proBNP **(D)**, Adiponectin **(E)**, MR-proANP **(F)**, and MR-proADM **(G)** regard to low, normal and above normal BMI. Median plasma levels of NT-proBNP **(D)**, Adiponectin **(E)**, MR-proANP **(F)** were significantly elevated in the patients with low BMI (p < 0.05 for trend).

The univariable linear correlation between BMI and adiponectin, NT-proBNP, MR-proANP, MR-proADM, copeptin, α-defensins, and hsCRP concentrations was evaluated. A significant inverse relationship was found between BMI and adiponectin (β = −0.42), NT-proBNP (β = −0.33), and MR-proANP (β = −0.19) levels. We did not find any association with the other biomarkers examined. Plasma levels of biomarkers according to BMI categories are shown in Figure 1 A-F. Multivariable linear regression analyses were performed for each biomarker, adjusted for age, sex, LVEF, NYHA, eGFR, and DM (base model). An independent impact of BMI on NT-proBNP (β = −0.32) and adiponectin (β = −0.39) levels in plasma was observed. BMI did not correlate to α-defensins, MR-proANP, MR-proADM or copeptin (Table 2). When eliminating age from the multivariable linear regression analysis with MR-proANP, this biomarker remained independently associated with BMI.

**Table 2:** Multivariable linear regression analyses with body mass index (BMI)

	**Dependent variable: BMI**	
Variables	Standardized coefficient β (SE)	P
Adiponectin	−0.39 (0.07)	<0.001
NT-proBNP	−0.32 (0.07)	<0.001
MR-proANP	−0.11 (0.08)	0.16
MR-proADM	0.05 (0.08)	0.57
Copeptin	−0.12 (0.07)	0.13
α-defensins	−0.06 (0.07)	0.46
hsCRP	0.01 (0.09)	0.89

All the respective biomarker-models are adjusted for age, sex, LVEF, NYHA, eGFR, andT2D.

*Abbreviations*: eGFR: estimated glomerular filtration rate; LVEF: left ventricular ejection fraction; MR-proADM: mid-regional pro-adrenomedullin; MR-proANP: mid-regional pro-A-type natriuretic peptide; NT-proBNP: N-terminal pro-B-type natriuretic peptide; NYHA: New York Heart Association functional classification; T2D: type 2 diabetes.

## Discussion

We have previously reported that BMI has an effect on plasma NT-proBNP concentrations in CHF [6]. In the current study we extended these findings on NPs to include MR-proANP, reporting that concentrations of both NT-proBNP and MR-proANP are negatively associated with BMI. After adjusting for parameters with known impact on concentrations of NPs, levels of NT-proBNP remained independently associated with BMI. By contrast, the other biomarkers examined reflecting neurohormonal stimulation, inflammation and endothelial dysfunctions were not related to BMI in the present CHF population.

Low BMI is associated with an unfavourable survival in CHF and accordingly it may be hypothesized that biomarkers significantly associated with BMI are mechanistically linked to the weight loss. In line with this the interest in the lipolytic role of natriuretic peptides is expanding. Recently, NPs have been shown to enhance lipolysis of adipose tissue though activation of the hormone sensitive lipase [26]. Furthermore, in experimental studies of ANP and BNP, an increase in energy utilization and thermogenesis by enhancing ‘browning’ of white adipocytes has recently been reported [3], hence, suggesting a pathophysiological explanation of the inverse relationship between BMI and NPs. We found a gradual decline in plasma MR-proANP levels corresponding to an increase in BMI, which is in accordance with findings of a previous study by Masson et al. [27]. We did not however, observe an independent association between MR-proANP and BMI, after adjustment for age, gender and kidney function. This observation is in accordance with a study in healthy subjects, in whom kidney function and age appeared to be the primary predictors of MR-proANP [28]. Clearance mechanisms of MR-proANP and NT-proBNP are presumably not identical. Therefore, these findings support that secretion from the myocytes of A and B-type NPs are blunted in obesity [29].

In the present study decreasing BMI was associated with advanced age and when eliminating age from the base model, MR-proANP became independently related to BMI. A recent *post hoc* analysis from The BACH trial reported significant differences in MR-proANP levels between acute HF patients with highest vs. lowest BMI [8].

Atrial NP promotes adiponectin release in healthy subject [30]. This association is corroborated by the current study. More knowledge on this cross-talk between the heart and adipose tissue may be of importance in CHF.

The inverse association between adiponectin levels and BMI found in this study has been documented in both healthy populations and in cohort of CHF patients. Increased levels of adiponectin have been identified in patients with extremely low BMI as in cardiac cachexia [7], a syndrome present in approximately 10% of a CHF population [5]. In this context, it is interesting that high plasma concentrations of adiponectin in the more advanced state of CHF are independent of BMI [12]. On this basis it has been suggest that adiponectin contributes to weight loss in cardiac cachexia by increasing energy expenditure [31].

Chronic low grade inflammation is involved in the pathophysiology of CHF. We have recently reported that elevated levels of the novel biomarker α-defensins, reflecting the innate immune system, have prognostic implications in CHF patients [18]. Plasma levels of α-defensins were not affected by BMI in the present study and this finding indicate that the innate immune system is not directly linked to the progressive weight loss observed in CHF with cachexia.

Circulating concentrations of biomarkers reflecting low-grade inflammation and hsCRP, endothelial dysfunction, MR-proADM, and water homeostasis copeptin were not associated with BMI in the present study, even though hsCRP concentrations were increased by a factor 2 in CHF with BMI < 21 kg/m^2^. In contrast to our findings, recent data on MR-proADM levels in lean as well as obese individuals demonstrated a positive correlation to BMI, with a progressive decrease in plasma levels during weight reduction after gastric by-pass surgery [14,28]. None of the novel biomarkers affected the association between BMI and outcome.

There are some limitations to this study. First, there are a limited number of patients enrolled in the current study, which might diminish the statistical power of detecting associations between BMI and biomarkers. Second, this study was monocentric and only BMI and no other anthropometric data were available in this CHF cohort. Finally, it should be noted that the present analyses are post hoc analyses on data collected for other purposes [6]. This may increase the risk for a Type I error. Whether we have overlooked a small effect of BMI on α-defensins due to a low sample size (a Type II error) can neither be excluded and our findings should be confirmed in larger cohorts.

## Conclusions

Among the biomarkers which have been shown to be of prognostic importance in patients with CHF only NPs and adiponectin were associated with BMI, as concentrations of the biomarkers increased by decreasing BMI. As this is a cross sectional study, our data do not allow us to make any conclusions about causality, but nevertheless, we speculate that these peptides may participate in the mechanisms responsible for the accelerated weight loss seen in patients with severe CHF.

## Competing interests

The authors declare that they have no competing interests.

## Authors’ contributions

CK, JF, MS, and HMC participated in the design of this study. HMC, CK, MS, and JF performed the statistical analyses. HMC, CK, JF, and MS drafted the manuscript. CK, MS, JF, JPG, JFR, and AF were involved in data collection and/or made important intellectual contributions to the interpretation of data and the writing of paper. All authors critically revised and approved the final version.

## Pre-publication history

The pre-publication history for this paper can be accessed here:

http://www.biomedcentral.com/1471-2261/13/80/prepub
